# New approach to assess sperm DNA fragmentation dynamics: Fine-tuning mathematical models

**DOI:** 10.1186/s40104-017-0155-7

**Published:** 2017-03-07

**Authors:** Isabel Ortiz, Jesús Dorado, Jane Morrell, Jaime Gosálvez, Francisco Crespo, Juan M. Jiménez, Manuel Hidalgo

**Affiliations:** 1 Veterinary Reproduction Group, Department of Animal Medicine and Surgery, Faculty of Veterinary Medicine, University of Cordoba, 14071 Cordoba, Spain; 2 Department of Clinical Sciences, Division of Reproduction, Swedish University of Agricultural Sciences, Box 7054, SE-75007 Uppsala, Sweden; 3 Department of Biology, Universidad Autónoma de Madrid, 28049 Madrid, Spain; 4 Department of Reproduction, Centro Militar de Cría Caballar (FESCCR-Ministry of Defense), 05005 Ávila, Spain; 5 Department of Chemistry and Physics of Materials, University of Salzburg, Hellbrunner Straße 34/III, A-5020 Salzburg, Austria

**Keywords:** Colloid centrifugation, Dynamics, Fine-tuning, Mathematical models, Sperm DNA fragmentation

## Abstract

**Background:**

Sperm DNA fragmentation (sDF) has been proved to be an important parameter in order to predict in vitro the potential fertility of a semen sample. Colloid centrifugation could be a suitable technique to select those donkey sperm more resistant to DNA fragmentation after thawing. Previous studies have shown that to elucidate the latent damage of the DNA molecule, sDF should be assessed dynamically, where the rate of fragmentation between treatments indicates how resistant the DNA is to iatrogenic damage. The rate of fragmentation is calculated using the slope of a linear regression equation. However, it has not been studied if sDF dynamics fit this model. The objectives of this study were to evaluate the effect of different after-thawing centrifugation protocols on sperm DNA fragmentation and elucidate the most accurate mathematical model (linear regression, exponential or polynomial) for DNA fragmentation over time in frozen-thawed donkey semen.

**Results:**

After submitting post-thaw semen samples to no centrifugation (UDC), sperm washing (SW) or single layer centrifugation (SLC) protocols, sDF values after 6 h of incubation were significantly lower in SLC samples than in SW or UDC. Coefficient of determination (R^2^) values were significantly higher for a second order polynomial model than for linear or exponential. The highest values for acceleration of fragmentation (aSDF) were obtained for SW, followed by SLC and UDC.

**Conclusion:**

SLC after thawing seems to preserve longer DNA longevity in comparison to UDC and SW. Moreover, the fine-tuning of models has shown that sDF dynamics in frozen-thawed donkey semen fit a second order polynomial model, which implies that fragmentation rate is not constant and fragmentation acceleration must be taken into account to elucidate hidden damage in the DNA molecule.

## Background

The importance of the assessment of sperm chromatin to predict the potential fertility has been shown in humans and animals [1–5]. The crucial role that sperm DNA fragmentation (sDF) plays in sperm analysis is due to its relationship with infertility problems in spite of apparently normal values for routine sperm parameters such as motility, morphology or integrity of sperm membranes [6]. The assessment of this parameter is even more critical when sperm quality is limited or compromised, as happens in some subfertile males, or in cool-shipped or frozen-thawed semen samples [7]. Therefore, it becomes of the utmost importance to select spermatozoa with intact DNA in order to achieve a higher success in pregnancy rates [8].

Previous studies have evaluated the effect of different centrifugation techniques to select frozen-thawed donkey sperm [9, 10] concluding that, although sperm quality was improved when colloid centrifugation was performed, this procedure did not select intact DNA spermatozoa when performing a static analysis of sDF (baseline value). Nevertheless, it has been shown in several studies [11, 12] that a dynamic assessment of sDF is more accurate to simulate ex vivo sperm maintenance and to evaluate latent chromatin damage than only considering baseline values. In these studies semen samples were submitted to thermal stress and sDF values were recorded at different times. Subsequently, a linear regression equation was calculated and the rates of fragmentation (the slope of the linear regression equation, sDF%/time) of the treatments were compared.

This approach solved the issue of the cryptic DNA damage; however, another question arose: do DNA dynamics fit to a linear regression model? Although linear regression is the simplest model, it entails that DNA damage is a simple process with constant speed. Before accepting this statement as an actual fact, a fine-tuning of mathematical models for DNA fragmentation over time should be carried out. Thus, the objectives of this study were to evaluate the effect of different after-thawing centrifugation protocols on sperm DNA fragmentation and elucidate the most accurate mathematical model for describing DNA fragmentation dynamics in frozen-thawed donkey semen.

## Methods

### Animals and semen collection

Six healthy Andalusian donkeys (aged from 6 to 15) were used for this study. Semen collection was performed using a Missouri artificial vagina with an in-line gel filter (Minitüb, Tiefenbach, Germany) in the presence of a jenny in natural or induced estrus. Three ejaculates per animal were collected, obtaining a total number of 18 ejaculates. All animal procedures were performed in accordance with the Spanish laws for animal welfare and experimentation.

### Sperm freezing and thawing

Sperm was frozen and thawed following the methodology described by Ortiz et al. [10]. Briefly, seminal plasma was removed by centrifugation (400 × *g* for 7 min) and the sperm pellet was resuspended with a commercial freezing medium containing egg-yolk and glycerol (Gent; Minitüb, Tiefenbach, Germany). Then, semen was slowly cooled for 2 h, loaded into 0.5 mL straws, placed 2.5 cm above the surface of the liquid nitrogen (LN_2_) for 5 min and plunged in LN_2_. Thawing was performed in a water bath at 37 °C for 30 s.

### Sperm processing after thawing

After thawing, each semen sample was divided into three aliquots and submitted to different centrifugation protocols.

#### Uncentrifuged diluted control (UDC)

After thawing, sperm was extended in a physiologically balanced solution that would support sperm viability (INRA96; IMV Technologies, L’Aigle, France) to a final concentration of 25×10^6^ sperm/mL. Sperm parameters were analyzed as described below.

#### Sperm washing (SW)

Sperm was thawed, diluted at the 1:1 ratio and centrifuged (400 × g for 7 min). The sperm pellet was resuspended in INRA96 to 25×10^6^ sperm/mL for sperm analysis.

#### Single layer centrifugation (SLC)

Sperm selection was carried out using the colloid Androcoll-E-Small (Swedish University of Agricultural Sciences, Uppsala, Sweden) as described by Ortiz et al. [10]. In short, 2 mL of thawed sperm were carefully placed on 4 mL of colloid. The suspension was centrifuged (300 × g for 20 min) and the pellet was resuspended in INRA96 to a final concentration of 25×10^6^ sperm/mL. Then, sperm parameters were assessed as described in the following.

### Sperm analysis after thawing

#### Sperm motility and membrane integrity

Total (TM, %) and progressive (PM, %) sperm motility were evaluated by computer-assisted sperm analysis (CASA) using the Sperm Class Analyzer (SCA 2011 v.5.0.1; Microptic S.L., Barcelona, Spain) with the settings described by Ortiz et al. [13]. Membrane integrity was assessed using Vital Test kit (Halotech DNA, Madrid, Spain) following the manufacturer’s instructions [10]. Red-stained sperm were considered as membrane-damaged and green sperm were considered as membrane-intact sperm (MIS, %).

#### Sperm DNA fragmentation (sDF) analysis

The degree of DNA damage in each sample was quantified using the sperm DNA fragmentation index. This parameter was assessed using the Halomax kit (Halotech DNA SL, Madrid, Spain) as previously described by Ortiz et al. [10]. This test is based on the dispersion of the chromatin (halo) after an exposure to a lysing solution. In order to evidence the halos of chromatin, samples were stained with a commercial kit for green fluorescence (Halotech DNA SL). Those sperm with large halos (at least double diameter than the core) were considered to have fragmented DNA. At least 300 spermatozoa per sample were counted and the percentage of fragmented DNA cells was recorded (sDF, %).

### Experimental design

#### Experiment 1: Effect of UDC, SW and SLC on DNA fragmentation dynamics

A dynamic assessment of DNA fragmentation was carried out by incubating an aliquot from UDC, SW and SLC samples for 24 h at 37 °C. The sDF was evaluated at T0 (baseline), T3, T6 and T24 h and compared between and within treatments.

#### Experiment 2: Comparison between the coefficient of determinations (R^2^) of linear, exponential and polynomial regression in sDF dynamics

The accuracy of three different regression models (linear, exponential, and polynomial) was evaluated by comparing the coefficient of determination (R^2^). Then, sDF dynamics were compared among treatments using the most accurate regression model.

### Statistical analysis

In Experiment 1 statistical analysis was performed using the Statistical Analysis Systems software (SAS v.9.0; SAS Institute Inc., Cary, NC, USA). A general linear model (PROC MIXED) with animals, ejaculates, treatments and time as fixed effects was performed. Differences between treatments (UDC vs. SW vs. SLC) and times (T0 vs. T3 vs. T6 vs. T24) were assessed.

In Experiment 2, sDF (%) values (*y* coefficient) at 0, 3, 6 and 24 h (*x* coefficient) were adjusted to linear, exponential and second order polynomial models. The R^2^ was calculated for each replicate and model using Microsoft Excel for Mac v.14 (Microsoft Corporation, Redmond, WA, USA) and R^2^ was compared separately for each treatment (UDC, SW and SLC), among models (linear, exponential and polynomial) with PROC MIXED (SAS) using animals and ejaculates as fixed effects.

Since second order polynomial functions are parabolic lines, the derivative function $\frac{d\  sDF}{ d t}\left(\frac{\%}{h}\right)$ was calculated for each treatment (UDC, SW and SLC). Afterwards, a graphic was represented using the rate of change of sDF (%/h, DNA fragmentation velocity) of the polynomial function $\frac{d\  sDF}{ d t}\left(\frac{\%}{h}\right)$ as *y* coefficient and time (0, 3, 6 and 24 h) as *x* coefficient. The slopes of these straight lines (DNA fragmentation acceleration, %/h^2^) were compared between treatments (UDC vs. SW vs. SLC) by ANCOVA using GraphPad Prism v.6 for Mac OS v.6 (GraphPad Software, San Diego, CA, USA).

All values were expressed as the mean ± standard error of the mean (SEM). Significant differences were considered when *P* < 0.05. Duncan *post hoc* test was carried out to assess differences between treatments.

## Results

### Sperm parameters after thawing

Mean values of sperm parameters obtained immediately after thawing were as follows: TM = 58.31 ± 4.57, PM = 47.66 ± 4.07, MIS =57.53 ± 2.71, and sDF = 12.98 ± 1.52.

#### Sperm DNA fragmentation (sDF) dynamics

**Fig. 1:**
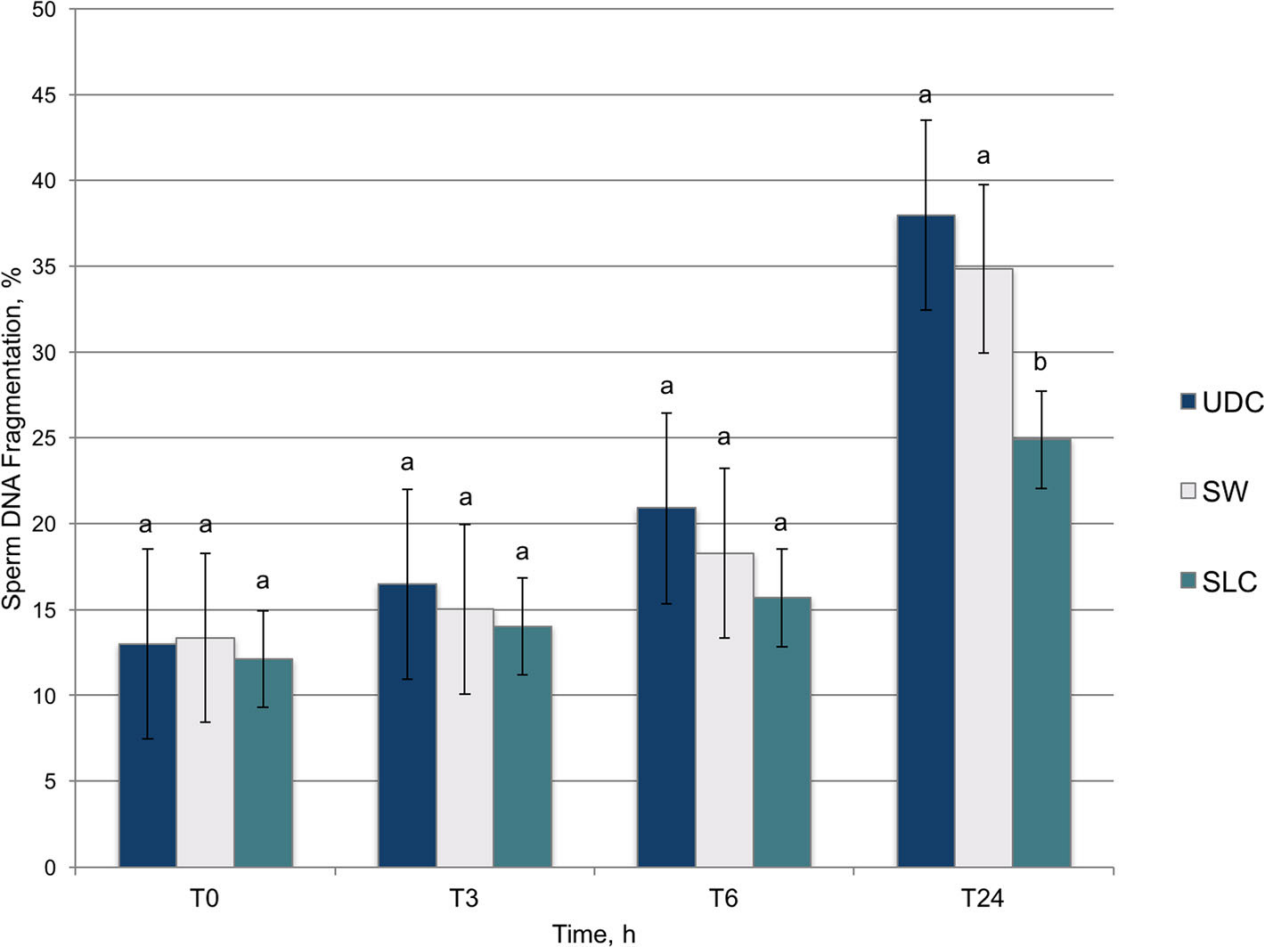
Effect of centrifugation (UDC, SW, and SLC) on the percentage of sperm DNA fragmentation (sDF, %) of frozen-thawed donkey sperm for 24 h of incubation at 37 °C. Values are expressed as means (*bars*) ± SEM (*error bars*). Different superscripts letters indicate significant differences (*P* < 0.05). UDC = Uncentrifuged diluted control; SW = Sperm washing; SLC = Single Layer Centrifugation; T0, T3, T6, T24 = Incubation for 0, 3, 6, 24 h at 37 °C

A comparison between UDC, SW, and SLC after thawing up to 24 h of incubation at 37 °C is shown in Fig. 1. Significantly lower values of sDF (*P* < 0.001) were found for SLC after 24 h of incubation.

Table 1 illustrates the sDF dynamics over time for each treatment (UDC, SW and SLC). Significantly higher values (*P* < 0.001) of sDF were obtained after 6 h of incubation for UDC and SW. However, in SLC-selected aliquots there were not significant differences for 24 h of incubation at 37 °C.

**Table 1:** Effect of time of incubation of sperm at 37 °C on sperm DNA fragmentation (sDF, %) within each centrifugation procedure (UDC, SW and SLC)

	T0	T3	T6	T24	*P*-value
UDC	12.98 ± 1.52^c^	16.47 ± 1.40^c^	20.90 ± 1.34^b^	35.86 ± 3.21^a^	<0.001
SW	13.35 ± 1.17^c^	15.01 ± 1.27^bc^	18.28 ± 2.21^b^	34.84 ± 2.60^a^	<0.001
SLC	11.48 ± 1.67^b^	14.02 ± 2.16^b^	15.70 ± 2.26^b^	24.91 ± 2.19^a^	<0.001

*T0, T3, T6, T24* incubation for 0, 3, 6, 24 h at 37 °C, *UDC* uncentrifuged diluted control, *SW* sperm washing, *SLC* single layer centrifugation

Values are expressed as mean ± standard error of the mean (SEM)

Different letters indicate significant differences

### Regression models fit to the data

**Fig. 2:**
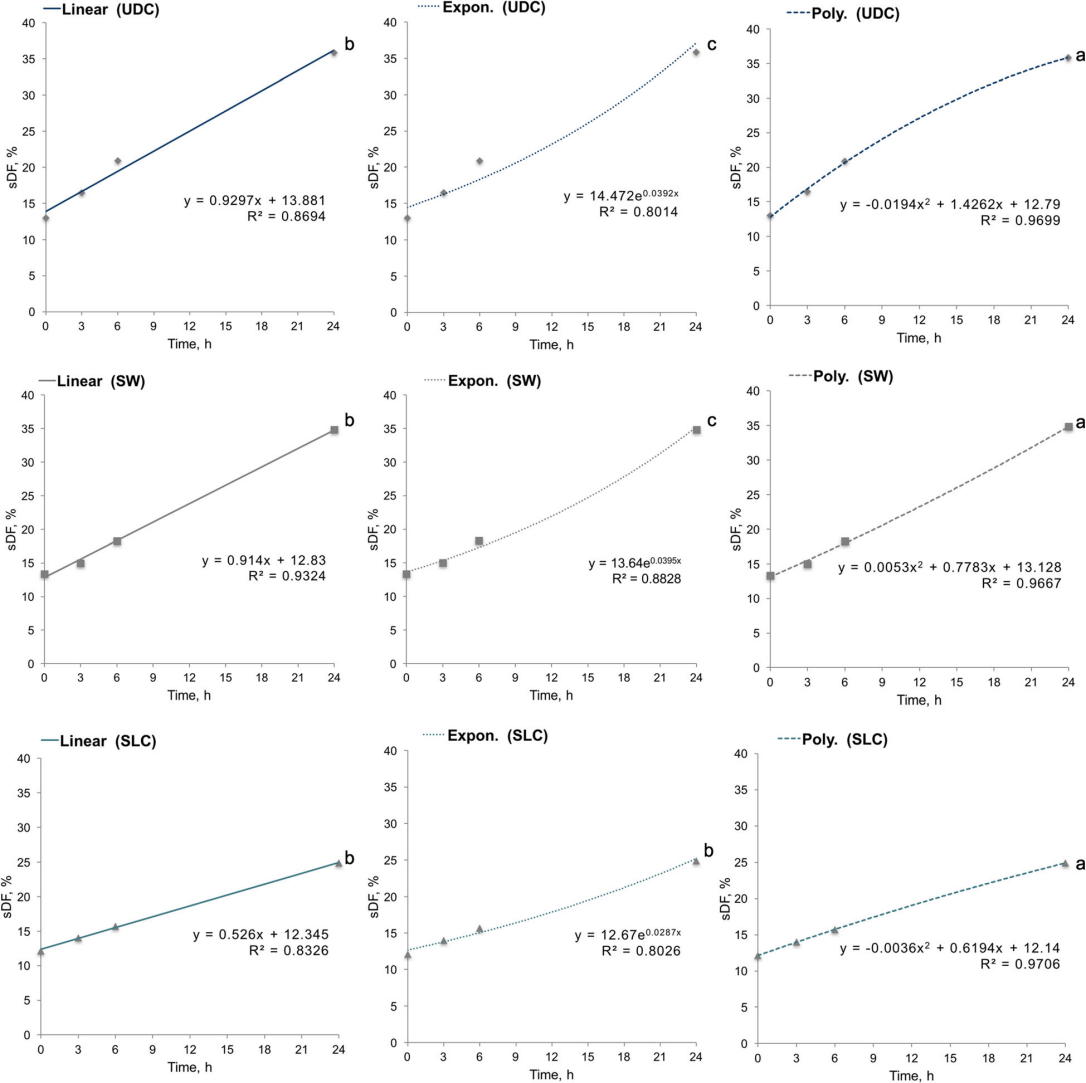
Coefficient of determination (R^2^) for linear, exponential and polynomial regression models within treatments (UDC, SW, SLC). Different letters indicate significant differences (*P* < 0.05). sDF = Sperm DNA fragmentation; UDC = Uncentrifuged diluted control; SW = Sperm washing; SLC = Single layer centrifugation; Expon. = Exponential; Poly. = Polynomial

The R^2^ for SDF dynamics was significantly higher (*P* = 0.001) in polynomial regression models in comparison to linear and exponential models for UDC (0.9699 ± 0.0087 vs. 0.8694 ± 0.0335 vs. 0.8014 ± 0.343), SW (0.9667 ± 0.0120 vs. 0.9324 ± 0.0190 vs. 0.8828 ± 0.0251), and SLC (0.9706 ± 0.0097 vs. 0.8326 ± 0.0605 vs. 0.0826 ± 0.0581) (Fig. 2).

### sDF dynamics in polynomial regression

**Fig. 3:**
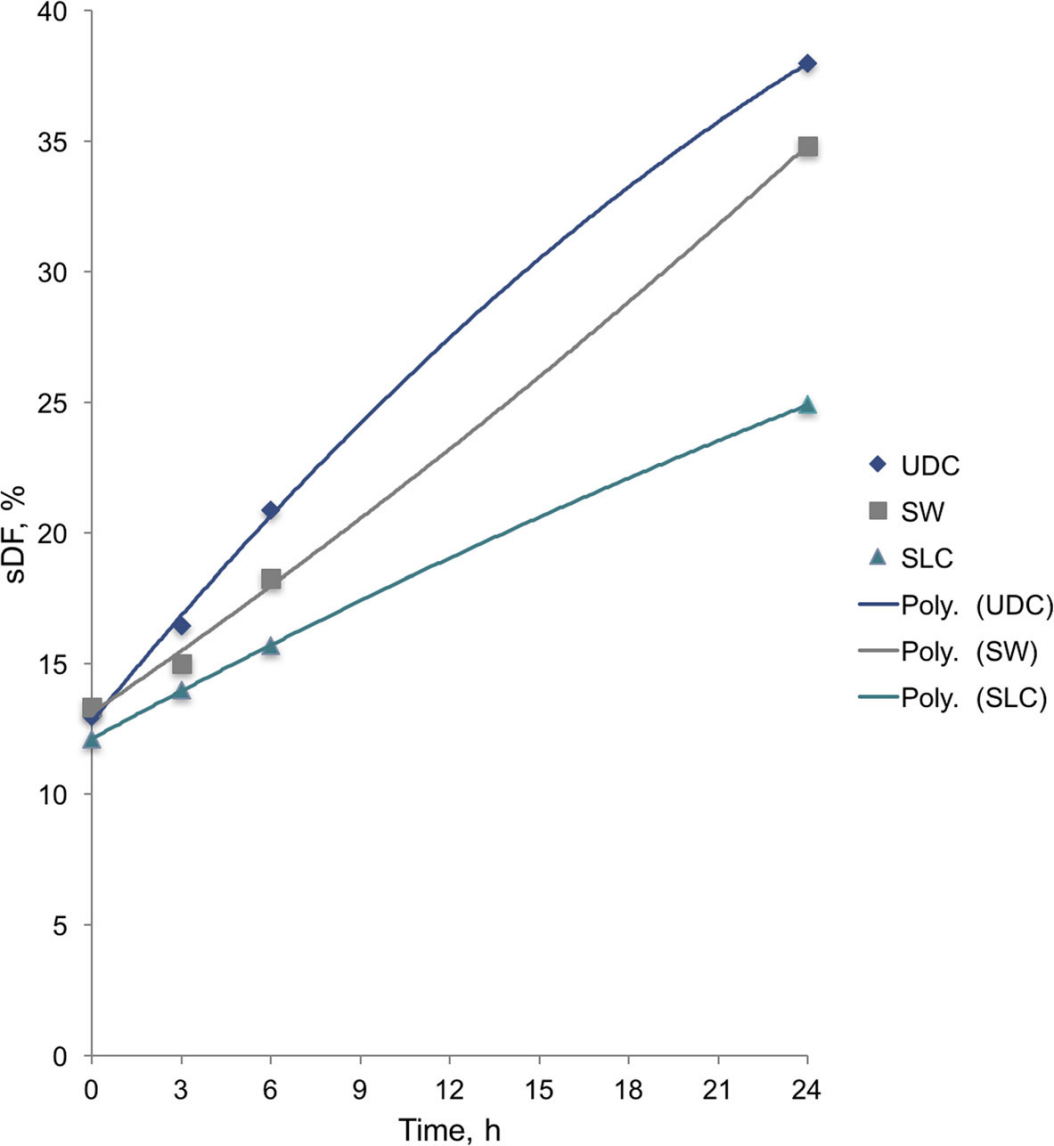
Polynomial regression lines for UDC, SW and SLC. sDF = Sperm DNA fragmentation; UDC = Uncentrifuged diluted control; SW = Sperm washing; SLC = Single layer centrifugation; Poly. = Polynomial

Figure 3 shows the graphical representation of the polynomial regression models for UDC, SW and SLC.

**Fig. 4:**
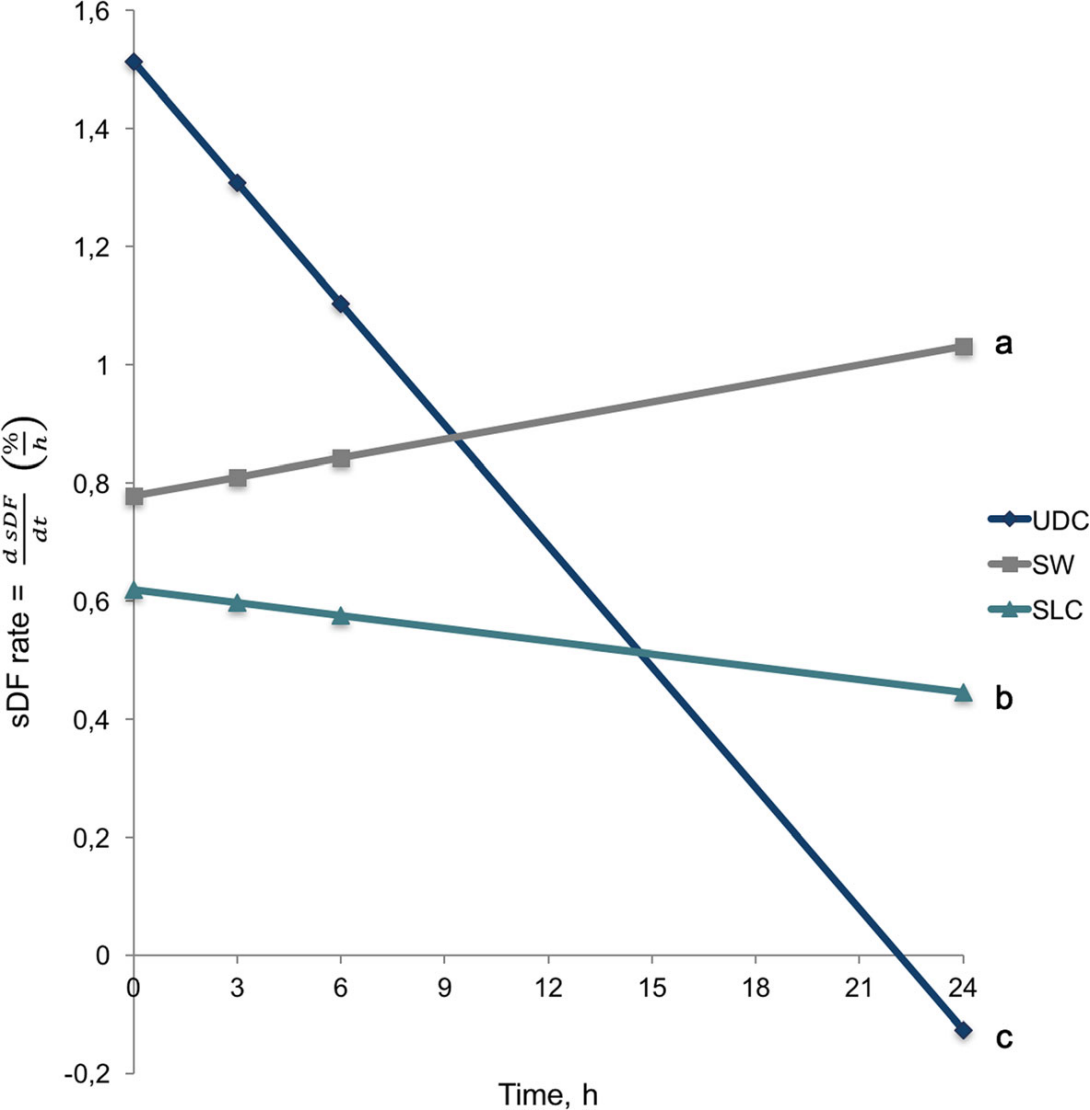
Sperm DNA fragmentation rate (%/h) in relation to time of incubation at 37 ºC for UDC, SW and SLC. Different letters indicate significant differences between slopes (*P* < 0.05). sDF = Sperm DNA fragmentation; UDC = Uncentrifuged diluted control; SW = Sperm washing; SLC = Single layer centrifugation

Since second order polynomial functions are graphically represented with parabolic lines, they cannot be compared with each other as a whole. The derivative of the polynomial function $\frac{d\  sDF}{ d t}\left(\frac{\%}{h}\right)$ is the rate of change of the function (fragmentation rate, %/h). Figure 4 represents the velocity of fragmentation with respect to time for UDC, SW and SLC. The slopes of these lines are the acceleration of fragmentation (aSDF, fragmentation rate/time, %/h^2^). Significant differences between the slopes of UDC (−0.0683 ± 0.0265), SW (0.0106 ± 0.0130) and SLC (−0.0073 ± 0.0141) were obtained (*P* = 0.0141).

## Discussion

The results of this study indicate that DNA in frozen-thawed donkey sperm selected by SLC is more resistant to a stressor (incubation at 37 °C up to 24 h) than control or SW. In order to compare sDF values obtained after each centrifugation procedure (UDC, SW and SLC), semen samples were submitted to incubation at 37 °C for 24 h. Afterwards, a static and a dynamic assessment of the DNA fragmentation dynamics were carried out. The static analysis of the sDF dynamics, which consisted of a comparison of treatments (UDC vs. SW vs. SLC) immediately finishing the centrifugation protocols (T0) and after 3 h (T3), 6 h (T6) and 24 h (T24) of incubation at 37 °C. No differences in sDF values were seen between treatments up to 24 h. The stability of the DNA molecule in each centrifugation protocol (T0 vs. T3 vs. T6 vs. T24) was evaluated. In this regard, sDF values did not increase until 6 h of incubation for UDC and SW. However, sDF remained stable for up to 24 h of incubation in SLC samples. Previous studies performed in stallion semen and donkey semen have not found differences in DNA fragmentation baseline values (T0) after colloid centrifugation [10, 14]. However, according to other studies, sDF must be studied dynamically by submitting the semen sample to a stressor in order to find possible cryptic damage in the DNA [11, 12, 15]. When a dynamic assessment was performed, other studies showed that SLC was able to select those stallion sperm more resistant to DNA fragmentation [16, 17]. Thus, the static and dynamic assessments of sDF dynamics seem to agree that the DNA fragmentation process in SLC samples is slower in comparison to UDC and SW.

Dynamic processes (such as sperm DNA fragmentation) are commonly used in biology as they provide insight into how a force (e. g. incubation at 37 °C over time) acts to change a cell, an organism, a population, or an assemblage of species [18]. Since we want to know how sDF changes over time *(t)*, that is, *sDF(t)*, a dynamic model is appropriate. There are two main types of dynamic models, “discrete time” and “continuous time”, depending on whether time is represented in discrete steps or along continuous axis. In practice, it is not possible to evaluate sDF continually: instead, we must divide the sample into aliquots at intervals of time. Thus, it is crucial to choose a suitable time scale for our study. Previous reports in donkeys [19] and horses [12] have shown that significant changes in sDF between treatments and individuals occurred from 6 to 24 h of incubation at 37 °C when using a chromatin dispersion test, or 4 h of incubation for the sperm chromatin structure assay (SCSA) by flow cytometry [20]. However, since one of the objectives of this study was to fine-tune a model, in order to adjust the model to reality, we needed to have as many points as possible. Therefore, we set the points at 0, 3, 6 and 24 h of incubation.

Once the variable (sDF) and the units of time (0, 3, 6 and 24 h) are stablished, their relationship was evaluated. The most common single-variable functions or mathematical models which regularly arise in all areas of biology are linear, exponential and polynomial. It is common to think of a relationship between two variables as a straight line; in fact, it has been taken for granted that sDF follows a linear regression equation

$$ y= f(x)= ax+ b; $$

where *y* = sDF (%), *x* = time(h); *a* = fragmentation rate (%/h); *b* = intercept (%). This model has been applied to calculate the sDF rate (rSDF) using the slope of the linear regression line. Nonetheless, a linear regression model, as its name indicates, is a straight line. This would mean that rSDF is constant over time which causes some limitations. In this sense, a phenomenon called the “Plateau effect” has been previously described when using linear regression to assess sDF dynamics in stallion sperm [12]. This so-called Plateau effect is a change in the slope of the regression line, due to a change in the velocity of fragmentation. This singularity leads to confounding results if the equation is not divided into two different lines, representing 0–6 h and 6–24 h of incubation. Nonetheless, that provisional adjustment shows that the sDF dynamics does not fit a linear regression equation.

Non-linear equations are those which can be represented as curves. In this sense, exponential and polynomial functions are very popular for modelling biological functions. Exponential function is probably one of the most important function in dynamic models of biology, it is represented as

$$ y= f(x)= b\cdot {e}^{ax}; $$

its main application is describing fast growth [21]. The exponential model has previously been applied to explain the behavior of sDF dynamics in human ejaculates [22]; nevertheless, this explanation was merely descriptive since there was no statistical comparison between other mathematical models. Last but not least, second order polynomial function (or quadratic),

$$ y= f(x) = a{x}^2+ b x+ c; $$

has a wide variety of important uses in biology. It describes the rate of growth when resources are limited [23]. Although previous studies have shown sDF curves which might fit a polynomial model [24, 25], to date, no study has tried to explained sDF dynamics using this model. Actually, to the best of the authors’ knowledge, a fine-tuning of models has never been performed for sDF dynamics. Furthermore, this quadratic model explains by itself the behavior of sDF from 0 to 24 h of incubation, without the need to split the 0–6 h and 6–24 h as it occurs with the traditional assessment by linear regression [12].

On the whole, mathematical models are mainly used to make predictions about the behavior of a variable at any time. Logically, the closer to reality the model is the more accurate the prediction will be. In statistics, R^2^ provides a measure of how well observed outcomes are replicated by the model, based on the proportion of total variation of outcomes explained by the model [26]. Surprisingly, second order polynomial achieved the significantly highest R^2^ in all the treatments studied (UDC, SW and SLC), becoming a more accurate model than linear regression or exponential to predict sDF over time. The more than acceptable R^2^ mean values obtained for UDC, SW and SLC (0.9699, 0.9667 and 0.9706, respectively) indicate that the conclusions obtained from this model are very close to reality.

The fact that our model is a parabola implies that sDF rate is not constant, which also means that there is sDF acceleration, an observation that has never been described before. In quadratic functions, we can track the rate of change of the function using a difference equation. A difference equation specifies how much a variable changes from one time unit to the next. In quadratic functions, the rate of change over time is expressed as follows:

$$ {y}^{\prime }= f^{\prime }(x)=\frac{dy}{dx}=2 ax+ b; $$

in our function, the rate of change of the function is the rate of change of rSDF. Therefore, sDF rate (rSDF) in quadratic functions is expressed as

$$ rSDF=\frac{dsDF}{dt}\left(\frac{\%}{h}\right), $$

and the fragmentation acceleration (aSDF) is the rate of change over time, or the slope of the derivative of the quadratic function:

$$ aSDF=\frac{dsDF/ dt}{t}\left(\frac{\%}{h^2}\right). $$

Surprisingly, when representing the rates of change for UDC, SW and SLC, the three lines obtained were very different. Although UDC showed faster rSDF for about 10 h, it was also the treatment with significantly lower acceleration (*aSDF*_*UDC*_ = −0.0683 ± 0.0265); this marked deceleration explains the Plateau effect described in stallions. In SW samples, rSDF values were lower than UDC until 10 h of incubation, but a higher acceleration (*aSDF*_*SW*_ = 0.0106 ± 0.0130) increased rSDF from that point on. The SLC also showed a negative acceleration (*aSDF*_*SLC*_ = −0.0073 ± 0.0141), but not as marked as in UDC samples. On the one hand, centrifuged samples after thawing (SW and especially SLC) showed lower values than control (UDC) for 10 and 15 h, respectively. On the other hand, centrifugation increased the acceleration of fragmentation, in particular in SW samples. It could be possible that post-thawing centrifugation, mainly SW, damaged the fixing mechanism of the DNA molecule [27, 28]. However, sDF values are lower during the time studied for SW and SLC. In this sense, if DNA fragmentation processes also occur in vivo in a non-linear manner, the initially rapid fragmentation rate may reduce the effective and fertile sperm concentration before they have a chance to colonize the oviducts. Therefore, delaying the early fragmentation events could be beneficial in this respect and may help to improve fertility. Further studies involving fertility and the relationship between DNA fragmentation timing and the timing of sperm transport and fertilization are needed to characterize the mechanism of action of this damage.

Nonetheless, we need to keep in mind that rSDF values have been obtained from a model, i.e. they are expected rather than observed values. In order to fit the model even more, further studies with more frequent assessments (between 6 and 24 h and after 24 h) would be needed to obtain more accurate predictions. It is of the utmost importance to fit data to an accurate model in order to know exactly how this molecule behaves. In this sense, DNA cannot have been correlated to any other sperm parameter [19, 29] using linear regression to work with sDF dynamics. Hopefully, this study provides new tips so that correlation between DNA fragmentation dynamics and sperm quality is focused from a new perspective.

## Conclusions

SLC after thawing seems to preserve DNA longevity for longer in comparison to UDC and SW. Moreover, the fine-tuning of models has shown that sDF dynamics in frozen-thawed donkey semen fits a second order polynomial model, which implies that fragmentation rate is not constant and fragmentation acceleration must be taken into account to elucidate hidden damage in the DNA molecule.

## Abbreviations

aSDF: Sperm DNA fragmentation acceleration
CASA: Computer-assisted sperm analysis
Expon.: Exponential
h: Hours of incubation at 37 °C
LN_2_: Liquid nitrogen
min: Minutes
MIS: Membrane intact sperm
PM: Progressive sperm motility
Poly.: Second order polynomial
R^2^: Coefficient of determination
rSDF: Sperm DNA fragmentation rate
s: Seconds
SAS: Statistical Analysis Systems Software
sDF: Sperm DNA fragmentation
SEM: Standard error of the mean
SLC: Single layer centrifugation
SW: Sperm washing
T0-T3-T6-T24: 0-3-6-24 h of incubation at 37 °C
TM: Total sperm motility
UDC: Uncentrifuged diluted control
